# Brain‐derived neurotrophic factor (BDNF) levels in panic disorder: A systematic review and meta‐analysis

**DOI:** 10.1002/brb3.3349

**Published:** 2023-12-31

**Authors:** Arman Shafiee, Kyana Jafarabady, Ida Mohammadi, Shahryar Rajai

**Affiliations:** ^1^ Department of Psychiatry and Mental Health Alborz University of Medical Sciences Karaj Iran; ^2^ Student Research Committee, School of Medicine Alborz University of Medical Sciences Karaj Iran; ^3^ School of Medicine Shahid Beheshti University of Medical Sciences Tehran Iran

**Keywords:** anxiety, brain‐derived neurotrophic factor, panic

## Abstract

**Background:**

The existing literature on the association between brain‐derived neurotrophic factor (BDNF) protein levels and panic disorder presents inconsistent findings. This systematic review and meta‐analysis aim to synthesize the available evidence and determine the overall effect of BDNF protein levels in individuals diagnosed with panic disorder.

**Methods:**

A comprehensive literature search was conducted using electronic databases (PubMed, Embase, Scopus, PsycINFO, and Web of Science) from inception to April 21, 2023. The search strategy included relevant keywords and medical subject headings terms related to BDNF, panic disorder, and protein levels. A random‐effects model was used for the meta‐analysis, and subgroup analyses were performed to explore heterogeneity. Publication bias was assessed using funnel plots and statistical tests.

**Results:**

A total of 12 studies met the inclusion criteria. The meta‐analysis demonstrated a significant decrease in BDNF protein levels in individuals with panic disorder (SMD = −.53, 95% CI: −1.02 to −.04, *p* < .001; *I* ^2^: 92%). The results of subgroup and meta‐regression analyses were not statistically significant. No significant publication bias was observed based on the results of Egger's regression test (*p*‐value = .3550).

**Conclusion:**

This systematic review and meta‐analysis provide evidence of lower BDNF protein levels in individuals diagnosed with panic disorder compared to healthy controls. The findings suggest a potential role for BDNF dysregulation in the pathophysiology of panic disorder. Further research is warranted to elucidate the underlying mechanisms and potential therapeutic implications.

## Graphical abstract

This systematic review and meta‐analysis provide evidence of lower BDNF protein levels in individuals diagnosed with panic disorders compared to healthy controls. The findings suggest a potential role for BDNF dysregulation in the pathophysiology of panic disorders. Further research is warranted to elucidate the underlying mechanisms and potential therapeutic implications.

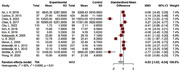

## 1 INTRODUCTION

Panic disorder is a prevalent and debilitating mental health condition characterized by recurrent and sudden panic attacks, often accompanied by intense fear and apprehension (Cackovic et al., 2022). These episodes are typically accompanied by physical symptoms such as palpitations, shortness of breath, chest pain, and a sense of impending doom (Cackovic et al., 2022). The etiology of panic disorder is multifactorial, involving complex interactions between genetic, neurobiological, and environmental factors (Perrotta, 2019).

Fear, the central component of panic disorder, is a complex of neuropsychological and behavioral events in response to the perception of dangerous stimuli (Musumeci & Minichiello, 2011). It has been observed that neurotrophins and especially brain‐derived neurotrophic factor (BDNF) play vital roles in the molecular pathways involved in fear learning and synaptic plasticity of the amygdala, as the manipulation of these factors in animal models has resulted in alterations in fear‐related responses (Choi et al., 2010; Dincheva et al., 2016; Psotta et al., 2013). Moreover, according to the neurotrophic theory of fear, neurotrophins are factors mediating the effects of past and recent experiences on the structure and function of fear pathway (Alleva & Francia, 2009), serving as the bedrock of individual variation in susceptibility to fear‐related disorders such as panic disorder.

BDNF is a neurotrophic, family of proteins that play a crucial role in the central nervous system's growth, development, and maintenance of neurons (Bathina & Das, 2015; Bekinschtein & von Bohlen und Halbach, 2020). It is primarily involved in neuronal survival, synaptic plasticity, and neurogenesis, making it a key player in various neurological and psychiatric disorders (Colucci‐D'Amato et al., 2020; Miranda et al., 2019). Several studies have explored the potential role of BDNF in anxiety disorders, aiming to elucidate its association with the pathophysiology of this condition (Farias et al., 2020).

The investigation of BDNF in panic disorder is motivated by its critical role in the development and function of the central nervous system (Li et al., 2022). It has been shown to decrease in panic disorder patients (Ströhle et al., 2010), with its reduction promoting development of panic disorder (Suliman et al., 2013). Polymorphisms reducing BDNF expression have also been linked to increased incidence of panic disorder (Xia et al., 2023), and treatments for panic disorder increase BDNF expression (Chen et al., 2017).

In humans, studies investigating BDNF protein levels in individuals with panic disorder have yielded mixed and inconsistent findings (Carlino et al., 2015; Li et al., 2023). Some studies have reported lower BDNF levels in individuals with panic disorder compared to healthy controls, suggesting a potential role for BDNF in the pathophysiology of the disorder (Chu et al., 2022). On the other hand, other studies have found no significant differences in BDNF levels between individuals with panic disorder and healthy controls (Carlino et al., 2015). These discrepancies may be due to variations in sample characteristics, measurement methods, or other confounding factors.

A systematic review and meta‐analysis offers an opportunity to synthesize the existing evidence, overcome the limitations of individual studies, and provide a more comprehensive understanding of the relationship between BDNF protein levels and panic disorder, amending the conflicting literatures. By aggregating data from multiple studies, we can increase statistical power, identify potential sources of heterogeneity, and explore subgroup analyses to uncover potential moderators or mediators of the BDNF‐panic disorder association. Therefore, we aimed to compare BDNF protein levels between panic disorder patients and healthy controls to validate its biomarker potential and further elucidate the underlying pathophysiology of panic disorder.

## 2 METHODS

### 2.1 Research question and objective

The primary objective of this systematic review and meta‐analysis is to examine the current evidence regarding BDNF levels in individuals diagnosed with panic disorder.

### 2.2 Study protocol and search strategy

A detailed study protocol was developed to guide the systematic review process and registered in PROSPERO with the following registration number (Table S1): CRD42023429158. The preferred reporting items for systematic reviews and meta‐analyses (PRISMA) guidelines were followed throughout the process (Page et al., 2021).

A comprehensive literature search was conducted across electronic databases including PubMed, Embase, Scopus, PsycINFO, and Web of Science. The search strategy combined relevant keywords and medical subject headings terms related to BDNF, and panic disorder. The search date was from the inception of the databases until April 21, 2023 (Table S2). In addition to the electronic search, manual searches of reference lists from relevant articles and review papers were performed to identify additional studies.

### 2.3 Study selection

Two independent reviewers conducted the study selection process in two stages: title/abstract screening and full‐text assessment. The reviewers resolved any disagreements through discussion, and a third reviewer was consulted if consensus could not be reached. Studies were included if they met the following criteria: (Cackovic et al., 2022) original research articles reporting BDNF levels in individuals diagnosed with panic disorder and had a control group; (Perrotta, 2019) studies conducted on human participants; and (Musumeci & Minichiello, 2011) studies providing sufficient data for effect size calculation or estimation. Studies were excluded if they were review articles, case reports, or conference abstracts publications.

### 2.4 Data extraction and quality assessment

A standardized data extraction form was created to collect relevant information from the included studies. Two independent reviewers extracted the following data: study characteristics (authors, publication year, and country), study design, sample size, participant characteristics (age, gender, drug free, and panic disorder diagnosis criteria), BDNF measurement tool, source (serum and plasma), panic disorder measures, age (mean + SD), and male (%). Any discrepancies were resolved through discussion, and a third reviewer was consulted if necessary.

The methodological quality and risk of bias of the included studies were assessed using appropriate tools, such as the Newcastle‐Ottawa Scale (NOS) for observational studies (Luchini et al., 2017).

### 2.5 Data synthesis and meta‐analysis

The meta‐analysis employed a random‐effects model to calculate the pooled effect size and assess its statistical significance. We used standardized mean difference (SMD) with its 95% confidence intervals (95% CIs) to report the pooled effect sizes. Subgroup and sensitivity analyses (using the leave one out method) were conducted to explore potential sources of heterogeneity and evaluate the robustness of the findings. Publication bias would be assessed using funnel plots and Egger's regression test.

## 3 RESULTS

### 3.1 Study selection

**FIGURE 1:**
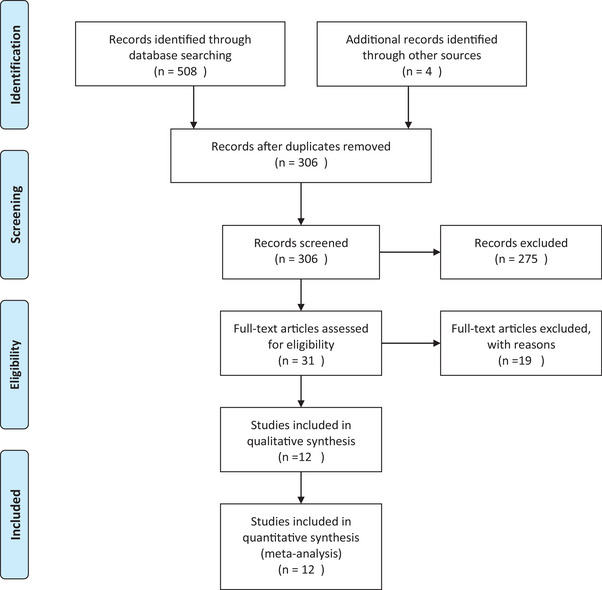
Preferred reporting items for systematic reviews and meta‐analyses (PRISMA) flow diagram.

A total of 508 articles were identified through electronic database searches, and an additional 4 articles were identified through manual searches. After removing duplicates, 306 articles underwent title and abstract screening. Of these, 31 articles were selected for full‐text assessment. Finally, 12 articles met the inclusion criteria and were included (An et al., 2019; Carlino et al., 2015; Chen et al., 2022, 2017; Chu et al., 2022; Kim et al., 2019; Kobayashi et al., 2005; Li et al., 2023; Maron et al., 2009; Molendijk et al., 2012; Ströhle et al., 2010; Wang et al., 2020) (see Figure 1 for the PRISMA flow diagram).

### 3.2 Study characteristics and quality assessment

The characteristics of the included studies are summarized in Table 1. The studies were published between 2005 and 2023 and were conducted in various countries, including China (*n* = 5), South Korea (*n* = 2), Italy (*n* = 1), Japan (*n* = 1), Germany (*n* = 1), Estonia (*n* = 1), and The Netherlands (*n* = 1). The sample sizes of the studies ranged from 60 to 457 participants, with a total of 2117 individuals included across all studies. At least 422 of the participants were drug free during BDNF sampling. The diagnosis of panic disorder was based on different criteria, including the Diagnostic and Statistical Manual of Mental Disorders (DSM‐V), DSM‐IV, and DSM‐III.

**TABLE 1:** Characteristics of included studies.

Author (year)	Country	Study design	BDNF measurement tool	Source (serum, plasma)	Number of participants	Panic definition	Drug free	Diagnostic tool for panic disorder	Age (mean + SD)	Male (%)
An et al. (2019)	South Korea	Case control	BDNF immunoassay System kit (Luminex, Austin)	Both	Cases = 52 Controls = 59 Total = 111	DSM‐IV	Yes	Korean version of the Mini International Neuropsychiatric Interview (MINI) by a trained psychologist	Case = 41.75 ± 14.08 Control = 38.47 ± 14.57	Case = 38.46 Control = 37.29
Carlino et al. (2015)	Italy	Case control	Emax Immuno assay System (Promega)	Serum	Cases = 37 Controls = 420 Total = 457	DSM‐IV‐TR	NA	Structured diagnostic interview using the Composite International Diagnostic Interview (SCID‐I)	Case = 48 Control = 47	Case = 28 Control = 52.14
Chen et al. (2022)	China	Case control	ELISA kits (R&D Systems)	Serum	Cases = 50 Controls = 50 Total = 100	DSM‐IV	No	Semistructured clinical interviews by two senior psychiatrists	Case = 40.60 ± 12.74 Control = 37.10 ± 12.43	Case = 40 Control = 36
Chen et al. (2017)	China	Case control	ELISA kit (DBD00, R&D Systems)	Serum	Cases = 30 Controls = 30 Total = 60	DSM‐IV	No	Hamilton anxiety rating scale (HARS) by two trained and experienced senior psychiatrists	Case = 35.50 ± 12.38 Control = 35.60 ± 12.90	Case = 50 Control = 53.33
Chu et al. (2022)	China	Case control	ELISA kit (DG10522H, Lvyuan Biotechnology)	Plasma	Cases = 116 Controls = 99 Total = 215	DSM‐5	Yes	Structured Clinical Interview for DSM‐5 (SCID‐5) AND Hamilton anxiety scale (HAMA‐14)	Case = 47.62 ± 10.56 Control = 49.95 ± 10.43	Case = 42.24 Control = 42.42
Kim et al. (2019)	South Korea	Case control	The BDNF Immunoassay System kit (Lumiplex, Australia reference)	Serum	Cases = 52 Controls = 59 Total = 111	DSM‐IV	Yes	Korean version of the Mini International Neuropsychiatric Interview (MINI)	Cases = 41.75 ± 14.08 Controls = 38.47 ± 14.57	Cases = 38.46 Controls = 37.29
Kobayashi et al. (2005)	Japan	Case control	The BDNF Emax immunoassay System kit (Promega)	Serum	Cases = 42 Controls = 31 Total = 73	DSM‐IV	No	Hamilton anxiety scale (HAMA‐14)	Cases: Good response = 31.5 ± 8.1 Poor response = 33.6 ± 9.2 Controls = 32.5 ± 12.6	Cases: Good response = 15.38 Poor response = 31.25 Controls = 16.13
Li et al. (2023)	China	Case control	Enzyme‐linked immunosorbent assay kits (DG10522H, Lvyuan Biotechnology)	Serum	Cases = 90 Controls = 99 Total = 189	DSM‐V	Yes	DSM‐5 diagnosis and Hamilton anxiety scale (HAMA‐14)	Cases = 47.62 ± 10.56 Controls = 49.95 ± 10.43	Cases = 38.9 Controls = 42.4
Maron et al. (2009)	Estonia	Cohort	ChemiKine BDNF Sandwich ELISA Kit (CHEMICON International, Inc.)	Serum	15 had panic attacks Total = 37	DSM‐III	Yes	Panic symptom scale (Panic attack defined as presence of four or more symptoms with sudden onset and at least moderate severity plus “fear/apprehension” item with a score three or four)	Total = 22.1 ± 4.2	Panic attack = 26.67 Total = 40.54
Molendijk et al. (2012)	The Netherlands	Case control	Emax Immuno Assay system from Promega	Serum	Cases with any type of anxiety disorder = 393 Cases (with panic disorder) = 97 Controls = 382	DSM‐IV	NA	Composite International Diagnostic Interview 2.1 (CIDI) life‐time version	Cases with any type of anxiety disorder = 41.0 ± 13.2 Cases with PD not provided Controls = 44.7 ± 12.3	Cases with any type of anxiety disorder = 33.3 Cases with PD not provided Controls = 38
Ströhle et al. (2010)	Germany	Case control	a modified fluorometric ELISA	Serum	Cases = 12 Controls = 12 Total = 24	DSM‐IV	Yes	The Mini International Neuropsychiatric Interview (MINI)	Cases = = 31.9 ± 7.62 Controls = 30.8 ± 8.66	Cases = 25 Controls = 25
Wang et al. (2019)	China	Case control	enzyme‐linked immunosorbent assay kits (DG10522H, Lvyuan Biotechnology)	Plasma	Cases = 85 Controls = 91 Total = 176	DSM‐V	Yes	Panic disorder severity scale–Chinese Version (PDSS‐CV) and Hamilton anxiety scale (HAMA‐14)	Cases = = 47.0 ± 10.8 Controls = 49.2 ± 10.5	Cases = 40.0 Controls = 42.9

The assessment of methodological quality and risk of bias indicated varying levels of quality among the included studies. The NOS was primarily used to evaluate the observational studies, with scores ranging from five to nine out of nine. Notably, none of the studies were found to have a high risk of bias (Figure S1).

### 3.3 Qualitative synthesis

The qualitative synthesis revealed heterogeneity in terms of study design, BDNF measurement methods, and panic disorder severity measures. Recent studies reported lower BDNF protein levels in individuals with panic disorder compared to healthy controls (Chu et al., 2022; Li et al., 2023; Wang et al., 2020). However, there were some discrepancies among the findings, with some studies reporting no significant differences (An et al., 2019; Carlino et al., 2015; Chen et al., 2022, 2017; Kim et al., 2019; Kobayashi et al., 2005; Molendijk et al., 2012) or even higher BDNF levels in individuals with panic disorder (Maron et al., 2009). Several studies also examined the relationship between BDNF levels and panic disorder severity, suggesting a potential association between lower BDNF levels and more severe symptoms.

### 3.4 Meta‐analysis

**FIGURE 2:**
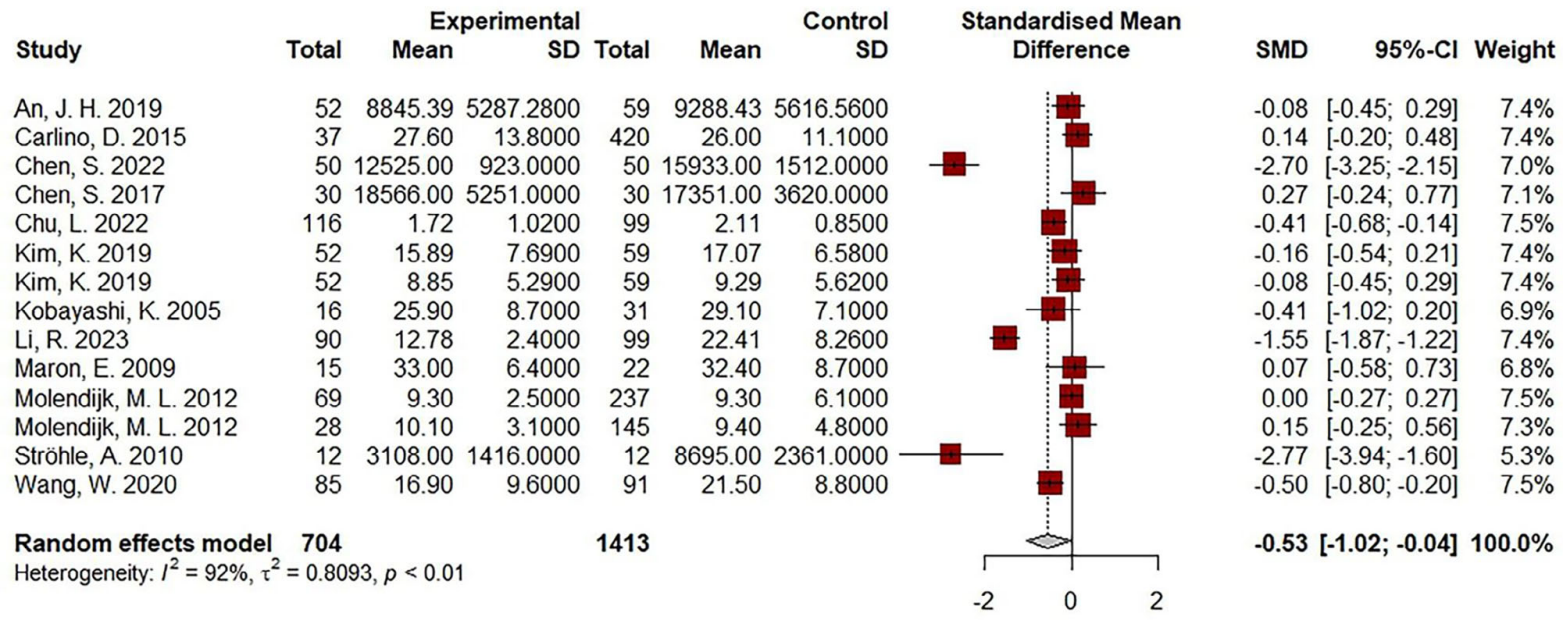
Results of meta‐analysis for the level of brain‐derived neurotrophic factor (BDNF) levels in panic disorder.

The meta‐analysis included 12 studies that provided sufficient data for effect size calculation with 704 cases and 1413 controls. The pooled effect size indicated a statistically significant decrease in BDNF levels in individuals with panic disorder compared to healthy controls (SMD = −.53, 95% CI: −1.02 to −.04, *p* < .001; *I* ^2^: 92%) (Figure 2).

### 3.5 Subgroup, meta regression, and sensitivity analyses

**FIGURE 3:**
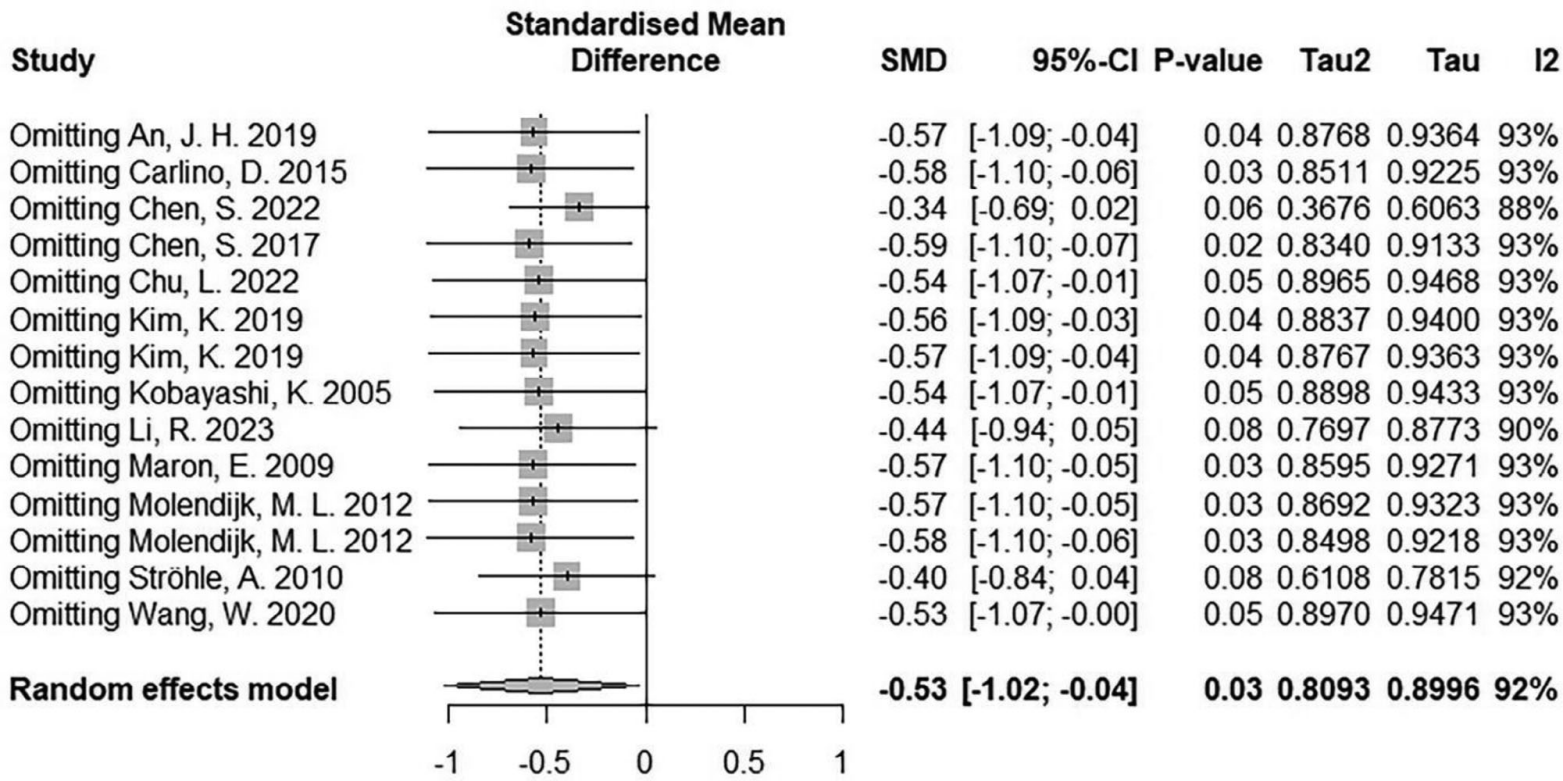
Results of sensitivity analysis.

Subgroup analyses were conducted to explore potential sources of heterogeneity. Subgroup analyses based on BDNF source, drug freedom of the sample, and panic disorder diagnosis criteria, did not show a significant between subgroup differences (Figures S2 and S3). Sensitivity analyses were performed by excluding one study at a time to evaluate the influence of individual studies on the overall effect size. The results of the sensitivity analyses showed that the stability of the findings was not achieved by removing several studies (Figure 3). Thus, the certainty regarding our findings may not be sufficiently high.

The results of our moderator analyses did not show a significant association between the pooled effect size and the following moderators: age (*p*‐value = 1.0000), male (*p*‐value = .7453), and year (*p*‐value = .4018).

### 3.6 Publication bias

**FIGURE 4:**
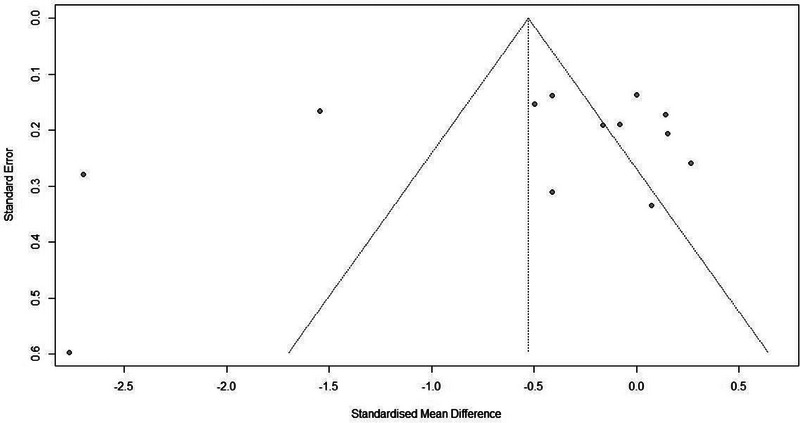
Funnel plot.

The funnel plot appeared symmetrical, indicating no significant publication bias. Egger's regression test also yielded nonsignificant results (*p* = .35), suggesting the absence of publication bias in the included studies (Figure 4).

## 4 DISCUSSION

The findings of our systematic review and meta‐analysis provide compelling evidence for lower BDNF levels among individuals with panic disorder. This observation is consistent with previous studies suggesting a potential involvement of BDNF in the pathophysiology of panic disorder (Suliman et al., 2013). The lower BDNF levels observed in panic disorder individuals may reflect dysregulation of the BDNF system, which could contribute to the development and maintenance of this disorder.

The role of BDNF in anxiety disorders has been previously validated by Suliman et al. (2013) by evaluating the results of 8 studies with the total sample size of 1179 patients. Their study examined data from 8 studies with a total sample size of 1179 patients. In line with our findings, their results demonstrated a significant decrease in BDNF levels among individuals with anxiety disorders. Additionally, their analysis revealed that BDNF levels obtained from serum were not statistically significant, whereas plasma levels showed significant differences. Consistent with these findings, our subgroup analysis examining the source of BDNF yielded similar results. Another systematic review and meta‐analysis have revealed that serum BDNF significantly decreases in patients who are diagnosed with anxiety disorders including post‐traumatic stress disorder and obsessive compulsive disorder (Suliman et al., 2013), yet panic disorder, a type of anxiety disorder (Hülya Kök, 2020), was not assessed in their work. Therefore, this evidence strengthens the hypothesis that BDNF levels decrease in panic disorder.

It is worth noting that a few studies have reported contradictory findings, with no significant differences in BDNF levels between individuals with panic disorder and controls (Carlino et al., 2015). Result of study done by Kim et al. (2019) showed nonsignificant differences in serum BDNF levels between panic disorder patients and healthy controls. However, these conflicting results may be attributed to various factors such as sample size, methodological differences, or the inclusion of different subtypes of panic disorder.

The role of BDNF in panic disorder can be understood within the context of its neurobiological functions. BDNF is involved in neuronal survival, synaptic plasticity, and neurogenesis, all of which are crucial for maintaining healthy brain function (10). BDNF is known to modulate synaptic plasticity, particularly in brain regions implicated in anxiety and fear, such as the amygdala and the hippocampus (Gorka et al., 2020; Miranda et al., 2019). Reduced BDNF levels in these regions may disrupt normal synaptic functioning and impair the regulation of emotional responses, leading to the manifestation of panic attacks (Phillips, 2017).

Furthermore, BDNF is involved in the regulation of the hypothalamic–pituitary–adrenal (HPA) axis, which plays a crucial role in the stress response (Naert et al., 2011). Dysregulation of the HPA axis has been implicated in panic disorder, with exaggerated stress responses contributing to the onset and maintenance of panic attacks (Abelson & Curtis, 1996). Lower BDNF levels could potentially disrupt the HPA axis regulation, leading to an increased vulnerability to stress and the development of panic symptoms (Suliman et al., 2013).

It is important to note that the relationship between BDNF and panic disorder is complex and likely influenced by various factors. Our meta‐analysis revealed a high degree of heterogeneity across the included studies, indicating potential sources of variability that could contribute to the observed differences in BDNF levels. Factors such as medication use, comorbidities, and disease duration may influence BDNF levels and should be considered when interpreting the findings.

Additionally, it is crucial to acknowledge the limitations of the current literature and the meta‐analysis conducted. The majority of studies included in our analysis were cross‐sectional in nature, limiting the ability to establish causality between BDNF levels and panic disorder. Longitudinal studies are needed to determine the temporal relationship and potential bidirectional effects between BDNF alterations and panic disorder. Moreover, methodological differences among studies, such as variations in sample characteristics, measurement techniques, and BDNF assessment methods, could contribute to the heterogeneity observed. Future research should aim to standardize methodologies and utilize more rigorous study designs to enhance the robustness and generalizability of the findings.

Despite these limitations, the consistent observation of lower BDNF levels among individuals with panic disorder provides important insights into the underlying neurobiology of this condition. It highlights the potential involvement of BDNF dysregulation in the pathophysiology of panic disorder and suggests that targeting BDNF pathways may hold promise for the development of novel therapeutic interventions (Deng et al., 2016).

The findings of this systematic review and meta‐analysis have clinical implications. If BDNF alterations are consistently associated with panic disorder, it could potentially serve as a biomarker for diagnostic purposes, aiding in the identification and differentiation of individuals with panic disorder. Additionally, understanding the role of BDNF in panic disorder may open new avenues for targeted therapeutic interventions. Modulating BDNF levels or its downstream signaling pathways could represent a novel approach to the treatment and management of panic disorder.

In conclusion, our systematic review and meta‐analysis demonstrate a significant association between lower BDNF levels and panic disorder. The findings support the notion that BDNF alterations may play a role in the neurobiology of panic disorder, potentially contributing to the dysregulation of anxiety‐related behaviors and fear responses. Further research is warranted to elucidate the mechanisms through which BDNF is involved in panic disorder and to explore its potential as a therapeutic target for this debilitating condition.

## AUTHOR CONTRIBUTIONS

**Arman Shafiee**: Conceptualization; project administration; data curation; writing—original draft; writing—review and editing; visualization. **Kyana Jafarabady**: Validation; resources; methodology; software; formal analysis; writing—original draft. **Ida Mohammadi**: Writing—original draft. **Shahryar Rajai**: Data curation

## CONFLICT OF INTEREST STATEMENT

The authors have no relevant affiliations or financial involvement with any organization or entity with a financial interest in or financial conflict with the subject matter or materials discussed in the manuscript.

## FUNDING INFORMATION

This study did not receive funding, grant, or sponsorship from any individuals or organizations.

### PEER REVIEW

The peer review history for this article is available at https://publons.com/publon/10.1002/brb3.3349.

## Supporting information

Table S1 PRISMA 2020 checklist.
Table S2 Search strategies for online databases.
Figure S1 Risk of bias assessment for each included study.
Figure S2 Subgroup analysis based on the source of sampling.
Figure S3 Subgroup analysis based on the diagnostic criterion of panic disorder.

## Data Availability

All data has been presented in the manuscript.
